# Isolated Rupture of the Teres Major Muscle When Water Skiing: A Case Report and Literature Review

**DOI:** 10.1155/2018/3806534

**Published:** 2018-04-01

**Authors:** Julien Cousin, Vincent Crenn, Alban Fouasson-Chailloux, Marc Dauty, Philippe Fradin, Francois Gouin, Guillaume Venet

**Affiliations:** ^1^Orthopedics and Trauma Department, University Hospital of Nantes, Nantes, France; ^2^Physical Medicine and Rehabilitation Center, University Hospital of Nantes, Nantes, France; ^3^Orthopedics and Trauma Department, Departmental Hospital Center of La Roche Sur Yon, La Roche-sur-Yon, France

## Abstract

Isolated lesions to the teres major muscle are rare. They generally occur in patients participating in sports such as baseball, tennis, or boxing. We report the case of a sports patient who suffered an isolated injury to the teres major while water skiing. The clinical presentation was confirmed by MRI. Conservative treatment was chosen and consisted of brief analgesic immobilization, followed by rehabilitative treatment. The rapid recovery of this patient with normal isokinetic strength evaluation at 6 months was interesting for objectifying full muscle recovery. Our results and the data from the literature suggest that functional rather than surgical treatment is preferable in isolated lesions to the teres major muscle.

## 1. Introduction

Isolated rupture of the teres major muscle is a rare condition; there is no real consensus in the literature on the treatment options, wavering between surgical and functional treatments, even though functional treatments seem to be a good option, with few risks. There are currently not enough objective measurements on strength recovery in medial rotation force following functional treatment. We propose the case report of an isolated teres major muscle rupture with functional treatment and isokinetic strength evaluation at 6 months in order to evaluate strength recovery precisely in this kind of lesion.

## 2. Case Presentation

The patient was a 39-year-old right-handed firefighter with no medical history. He consulted the emergency department for a violent pain that had occurred the same day as a result of pulling a cable while water skiing.

**Figure 1:**
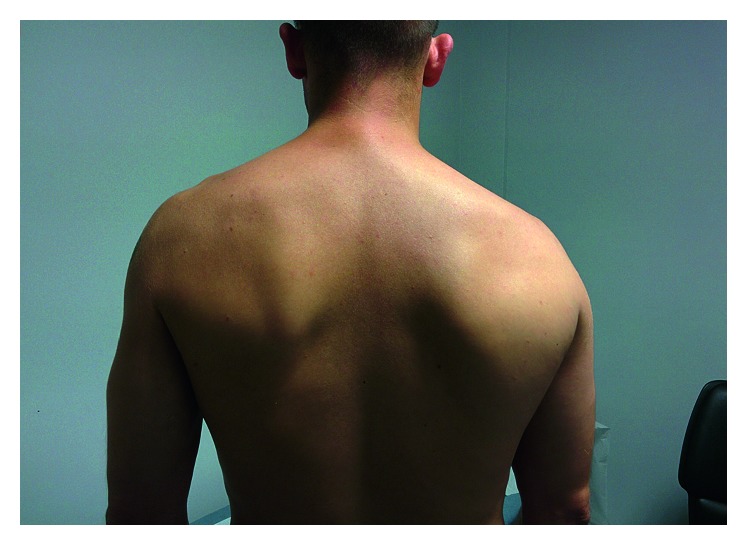
A 37-year-old patient with isolated rupture of the right teres major muscle. The photograph shows asymmetry with swelling at the lateral edge of the scapula.

The initial clinical examination revealed painful swelling at the lateral edge of the scapula (Figure 1). Active joint mobility was limited in all axes, while passive mobility was conserved. The neurological and vascular examination was normal. There was no sign of glenohumeral instability. Initial management consisted of analgesic immobilization with a standard arm sling in the adducted position for 2 weeks and the use of level 1 analgesics (paracetamol) and nonsteroidal anti-inflammatory drugs (ibuprofen).

**Figure 2:**
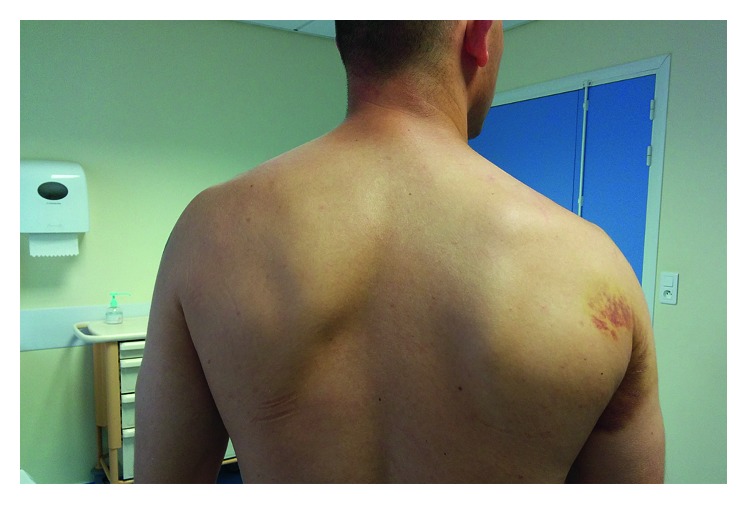
Presentation at 2 weeks: hematoma at the lateral edge of the scapula.

**Figure 3:**
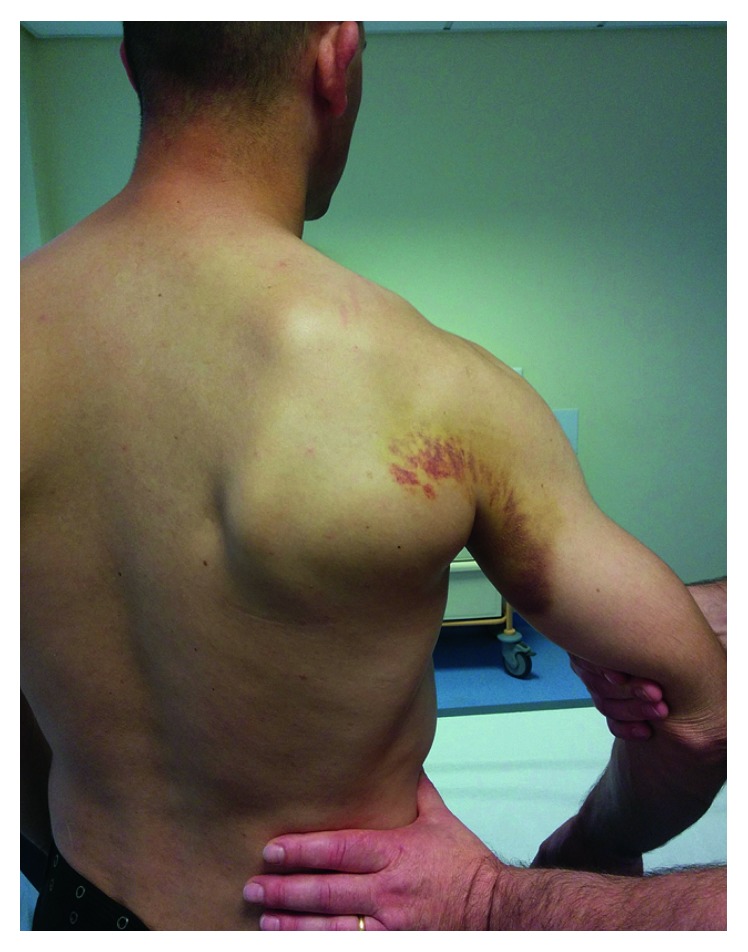
Presentation at 2 weeks: hematoma extending into the axilla and the medial aspect of the arm. Pain during flexion versus resistance of the teres major muscle.

The patient was seen at a 2-week trauma consultation for clinical reassessment and interpretation of his magnetic resonance imaging (MRI). The physical examination showed persistent edema at the lateral edge of the right scapula. Hematomas appeared next to the lateral edge of the scapula, continuing into the axilla and the medial part of the arm (Figures 2 and 3). Passive joint mobility was normal in all axes of mobility, with a loss of 10 to 20° in maximum amplitudes during active mobilization in abduction, antepulsion, and external rotation. Internal rotation was normal with 95° of mobility. The patient had no spontaneous pain (VAS = 0) but had mild pain with 4/10 on the VAS during isometric contraction of the teres major versus resistance. The muscular testing revealed a loss of internal rotation force during an adduction movement counterbalanced by support at the level of the arm evaluated at 4+/5.

**Figure 4:**
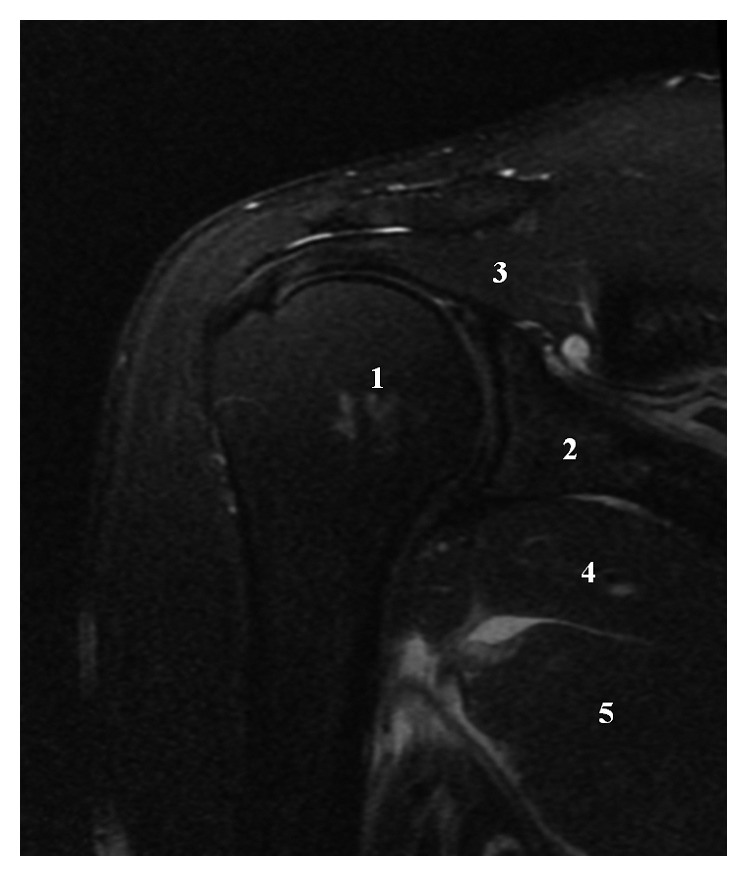
Coronal T2 MRI: (1) humeral head, (2) glenoid cavity, (3) supraspinatus muscle, (4) teres minor muscle, and (5) complete rupture of the teres major muscle.

**Figure 5:**
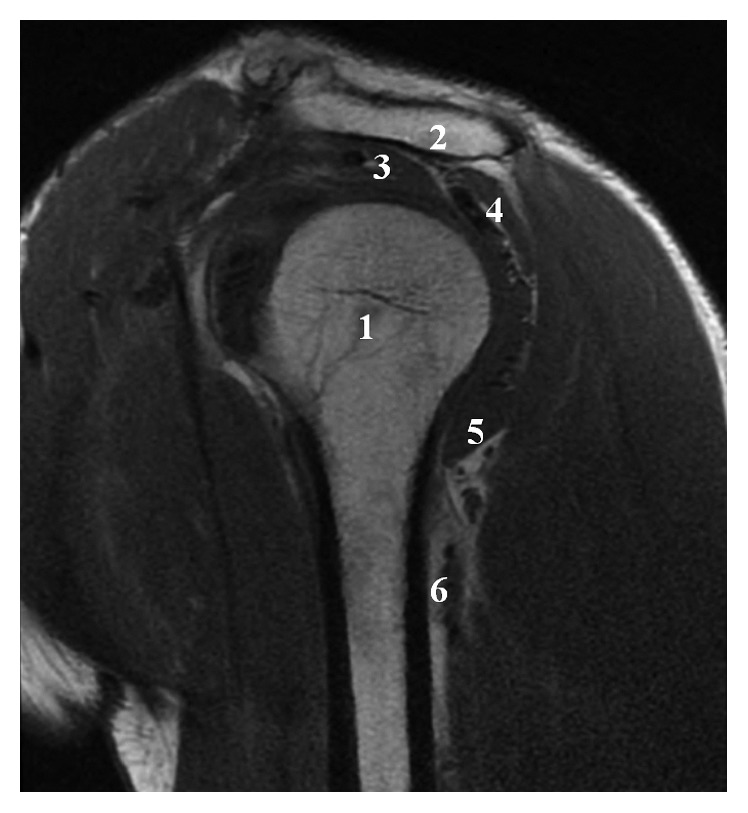
Sagittal T1 MRI: (1) humeral head, (2) acromion, (3) supraspinatus muscle, (4) infraspinatus muscle, (5) teres minor muscle, and (6) teres major rupture.

**Figure 6:**
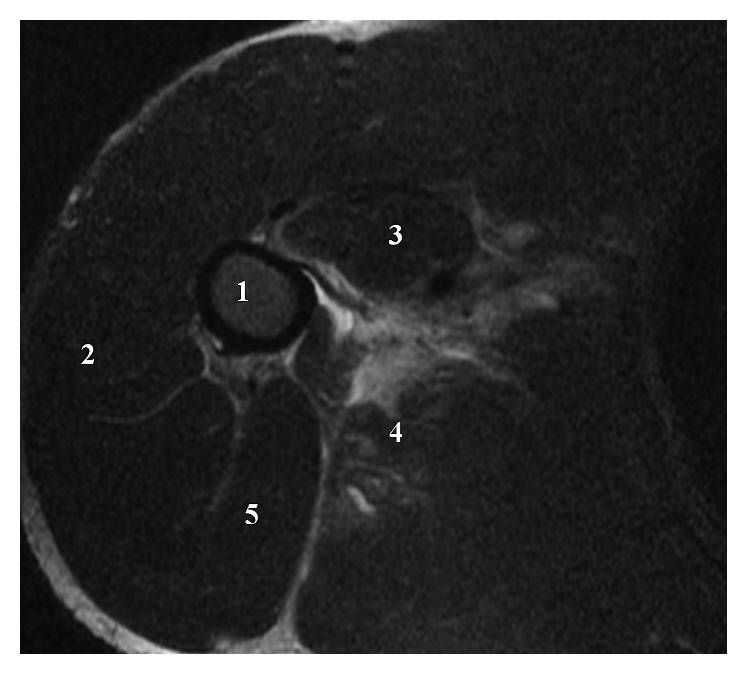
Axial T1 MRI: (1) humerus, (2) deltoid muscle, (3) coracobrachialis muscle, (4) teres major muscle, and (5) triceps muscle.

The MRI showed an isolated avulsion of the teres major muscle. There was no involvement of the latissimus dorsi muscle, nor of the teres minor muscle. The rest of the MRI was normal (Figures 4–6).

The management consisted in early rehabilitation. The rehabilitation protocol included physiotherapy for analgesic purposes, recovery of passive and active range of motion, followed by overall muscle reinforcement, and specific reinforcement of the teres major muscle in eccentric mode. Sick leave was also proposed for a period of 3 months in this patient, as his work requires daily physical activity. The evolution was quickly favorable according to the physiotherapist's follow-up to the reeducative management.

The patient was seen 6 weeks after the initial trauma at a trauma consultation. He described some residual pain in the posterior aspect of the proximal humerus. The clinical examination revealed a normal range of motion. He was able to intensify the rehabilitation.

At 6 months, the patient performed an isokinetic test, evaluating the teres major (medial rotator). The results showed an absence of strength deficit detected with an isokinetic dynamometer (Cybex Norm®, Ronkonkoma, NY, USA). Concentric peak torques were 53 nm on the right side versus 49 nm on the left at 60°/sec and 48 nm versus 47 nm at 120°/sec, which can be considered symmetrical. At that time, the patient was no longer complaining of either muscle weakness or pain.

## 3. Discussion

The teres major muscle originates on the dorsal aspect of the inferior angle and the lateral edge of the scapula, under the teres minor muscle. It follows an oblique course upwards and outwards to terminate on the medial side of the intertubercular groove of the humerus. It is innervated by the lower subscapular nerve, originating from the C5 and C6 roots. It plays a role in adduction, internal rotation, and retropulsion of the arm on the thorax, forming a single functional unit with the latissimus dorsi muscle.

Isolated lesions to the teres major muscle are rare. They are readily associated with lesions to the latissimus dorsi muscle given their anatomical proximity and their similar action during the practice of sports [1].

Lesions of the teres major muscle are thus the result of different types of traumatism: trauma in traction with the upper limb stretched, during eccentric concentration of the muscle that opposes the sudden traction. This mechanism often occurs in water skiing injuries, when the cable is suddenly tightened at start-up [2–5], at full speed in the throwing position, as in the case of baseball pitchers [6–9] and tennis players when serving [10].

The sports most frequently associated with this type of injury are baseball [6–9] and water skiing [2–5]. Rupture of the teres major muscle has also been reported in patients playing hockey [11], tennis [10], golf [12], and cricket [13], or those practicing boxing [14], as well as in a soccer goalkeeper [15].

When examining our patient, he told us that he felt a sudden tear as he pulled on the cable when starting from the platform. The eccentric contraction of the teres major, responsible for adduction and internal rotation and aimed at opposing the traction generated by the cable, was responsible for this isolated rupture of the teres major in our patient.

This clinical presentation seems similar for most authors. The patient felt severe pain with a tearing sensation in the axillary region associated with significant functional impotence.

The initial clinical examination found a swelling at the lateral edge of the scapula that could correspond to hematoma and muscle retraction. Active joint mobility was limited in all axes, while passive mobilization was normal. In general, the diagnosis can be confirmed using imaging tests: first-line MRI or ultrasound. Standard X-ray assessment makes it possible to search for bone removal and eliminate differential diagnoses. The therapeutic management is either functional or surgical, although this latter is rare in the literature.

Burks et al. reported a case in a 35-year-old SWAT patient who received surgical management after a combined rupture of the teres major and latissimus dorsi muscle [16]. The patient was immobilized with an arm sling during the postoperative period and underwent intensive rehabilitation from 6 weeks postoperatively. The evolution was favorable, and the patient returned to his professional activity without loss of strength.

Garrigues et al. reported the case of a 33-year-old patient with isolated teres major avulsion at the level of his humeral insertion [4]. He underwent surgical reinsertion with anchors followed by immobilization with an arm sling 6 weeks postoperatively. At 1-year, the patient had not noticed any functional disorder in his shoulder and the objective measurements were excellent. There was, however, a deficit in force of 27% in internal rotation and 24% in extension.

Naidu et al. reported 3 cases of professional cricket players [13] with avulsion of the latissimus dorsi and teres major muscles. Two underwent surgical repair with excellent results. The unoperated patient experienced discomfort until 7 months postrupture.

Other authors reported functional management associating short immobilization with an arm sling for analgesic purposes and progressive rehabilitation. The functional results reported by the authors are very satisfactory.

Lester et al. also reported the case of a 30-year-old patient injured when practicing this sport. He was not treated surgically, and at 3 months, he resumed his sports activities without any pain. He did not present any limitation in the articular amplitudes [3].

Fitzpatrick et al. presented the case of a 53-year-old patient who suffered an isolated rupture of the teres major muscle while water skiing, which was treated functionally [5]. He was able to return to his activities without limitations 14 weeks after the initial trauma.

Takase reported the case of a 21-year-old patient who presented this injury after serving in tennis [10]. He did not undergo surgery and was able to play tennis without restrictions at 6 months without sequelae.

Malcolm et al. reported the case of a 22-year-old baseball pitcher with an isolated rupture of the teres major muscle who received functional treatment [6]. Six months after the rupture, the patient had resumed his activity without pain or loss of performance.

Leland et al. described this lesion in 2 professional baseball players [7]. Neither underwent surgery, and both returned to the same level in the year following the trauma.

Schickendantz et al. reported a series of 10 cases in professional baseball players: 5 with isolated latissimus dorsi muscle rupture, 4 with isolated teres major lesions, and 1 with a combined lesion. All were treated functionally, and 9 were able to regain their level within 3 months of injury. There was 1 case of recurrence, and 1 patient had to withdraw from the season [8].

Grosclaude et al. reported 2 cases in professional hockey players. For one, the rupture occurred during the shoot phase and for the other, when striking the stick of an opponent during a face-off [11]. Both players were treated functionally and were able to play in less than a week.

Martin et al. described the first case of myotendinous disinsertion of the teres major and latissimus dorsi muscles in a professional boxer. He received functional management, and 4 weeks after the trauma, the clinical examination was normal. He was able to return to his activity at his previous level [14].

Regarding the absence of deficit, isokinetic strength evaluation was interesting as a means of objectifying muscle recovery. The isokinetic method is based on measuring muscle strength at a constant angular speed to estimate the relationship with an orthopedic or neurological disorder [17]. As reported in this case, the patient recovered totally after 6 months of a rehabilitation program.

The complete recovery of range of motion and strength in our patient, objectified by the isokinetic measurements at 6 months, favors functional treatment of an isolated teres major lesion. These data seem to be confirmed by the bibliographical analysis on this subject.

## 4. Conclusion

Isolated ruptures of the teres major muscle are rare but found in certain risky sports such as water skiing. In most cases, interrogation and clinical examination make it possible to suspect the diagnosis easily. Imaging is nevertheless necessary (MRI) to confirm the suspected diagnosis. Treatment based on adapted rehabilitation makes a quick return to physical activity possible without sequelae.
